# Real-World Assessment of Renal and Bone Safety among Patients with HIV Infection Exposed to Tenofovir Disoproxil Fumarate-Containing Single-Tablet Regimens

**DOI:** 10.1371/journal.pone.0166982

**Published:** 2016-12-12

**Authors:** Ella T. Nkhoma, Lisa Rosenblatt, Joel Myers, Angelina Villasis-Keever, John Coumbis

**Affiliations:** 1 Global Pharmacovigilance and Epidemiology, Bristol-Myers Squibb, Pennington, New Jersey, United States of America; 2 US Health Economics and Outcomes Research, Bristol-Myers Squibb, Plainsboro, New Jersey, United States of America; 3 Research and Development, Bristol-Myers Squibb, Plainsboro, New Jersey, United States of America; 4 Global Pharmacovigilance and Epidemiology, Bristol-Myers Squibb, Hopewell, New Jersey, United States of America; University of Pittsburgh, UNITED STATES

## Abstract

**Objectives:**

Tenofovir disoproxil fumarate (TDF)-containing antiretroviral regimens have been associated with an increased incidence of renal and bone adverse outcomes. Here, we estimated the real-world incidence of renal and bone adverse outcomes among patients with HIV infection receiving different TDF-containing single-tablet regimens (STRs).

**Methods:**

This cohort study used US health insurance data spanning the years 2008–2014. We identified HIV-infected patients aged ≥18 years (all HIV patients) and those with ≥6 months of continuous enrollment prior to initiating efavirenz/emtricitabine/TDF (EFV/FTC/TDF), rilpivirine/FTC/TDF (RPV/FTC/TDF) or elvitegravir/cobicistat/FTC/TDF (EVG/COBI/FTC/TDF). Renal adverse outcomes were identified using renal International Classification of Diseases, Ninth Revision, Clinical Modification (ICD-9-CM) diagnosis codes. Bone adverse outcomes were identified using ICD-9-CM diagnosis codes for fracture. Incidence rates (IRs) and associated 95% confidence intervals (CIs) were estimated assuming a Poisson distribution, and outcomes between STRs were compared using IR ratios (IRRs) and IR differences (IRDs).

**Results:**

We identified 9876 and 10,383 eligible patients for the renal and fracture analyses, respectively. Observed IRs for renal adverse outcomes were 9.7, 10.5, 13.6, and 18.0 per 1000 person-years among those receiving EFV/FTC/TDF, RPV/FTC/TDF, or EVG/COBI/FTC/TDF, or all HIV patients, respectively. Corresponding values for IRs of fracture were 3.4, 3.6, 7.2, and 4.4 per 1000 person-years, respectively. Renal adverse outcomes with EFV/FTC/TDF were significantly less frequent than with EVG/COBI/FTC/TDF (IRD −3.96; 95% CI: −7.31, −1.06). No IRR differences were identified for the renal analysis. Fractures with EFV/FTC/TDF were significantly less frequent than with EVG/COBI/FTC/TDF (IRR 0.47; 95% CI: 0.27, 0.81 and IRD −3.85; 95% CI: −5.02, −2.78).

**Conclusions:**

In this large real-world database, observed IRs for renal adverse outcomes with TDF-containing STRs were lower or similar to those for all HIV patients, with the lowest IRs observed among patients receiving EFV/FTC/TDF. Compared with all HIV patients, the observed IR for fracture was higher with EVG/COBI/FTC/TDF, comparable with RPV/FTC/TDF, and lower with EFV/FTC/TDF.

## Introduction

Antiretroviral therapy (ART) has led to dramatic improvements in survival among HIV-infected individuals [1], which has transformed a life-threatening disease into a chronic manageable condition requiring lifelong ART. Because patients with HIV infection are now living longer, they are increasingly affected by the common comorbidities of aging (e.g. cardiovascular disease, renal impairment and osteoporosis) [2]. Moreover, these comorbidities occur more often and at a younger age in patients with HIV infection compared with uninfected individuals [2]. Together with ART-associated toxicity potentially exacerbating these comorbidities and the increased risk of drug–drug interactions as patients age [2], these factors present additional challenges to physicians faced with choosing the most appropriate therapy for patients with HIV infection.

Single-tablet regimens (STRs) represent a significant advance in the management of HIV infection through simplifying ART, limiting the impact of therapy on daily life, and improving patient adherence to all components of their antiretroviral regimen [3].

Tenofovir disoproxil fumarate (TDF) is a common component of many multi-tablet antiretroviral regimens and is also included in 3 STRs: efavirenz (EFV)/emtricitabine (FTC)/TDF, rilpivirine (RPV)/FTC/TDF, and elvitegravir (EVG)/cobicistat (COBI)/FTC/TDF. Tenofovir is primarily eliminated via the kidney by a combination of glomerular filtration and active tubular secretion [4]. Systematic reviews and meta-analyses of randomized controlled trials have indicated an association between TDF-based regimens and an increased risk of renal impairment and bone demineralization [5,6,7], with longer-term studies suggesting that this risk is further increased with cumulative exposure to tenofovir [8,9,10,11].

TDF is subject to drug–drug interactions with a number of antiretroviral agents that increase tenofovir exposure, including ritonavir-boosted protease inhibitors [4,12], cobicistat-boosted regimens [13], and RPV [14]. However, there is no clinically significant pharmacokinetic interaction between EFV and TDF [15,16]. TDF is also subject to drug–food interactions that influence tenofovir exposure. Tenofovir bioavailability increases if TDF is administered with food, particularly after a high-fat meal [4,17]. Because EFV/FTC/TDF is recommended to be administered under fasting conditions [18], whereas RPV/FTC/TDF and EVG/COBI/FTC/TDF are administered with food [14,19], tenofovir exposure would be expected to be higher with these latter 2 STRs.

In a large, national sample of predominantly male individuals, cumulative exposure to EFV was associated with a lower risk of proteinuria and chronic kidney disease [10]

It is unclear whether these pharmacokinetic characteristics of EFV/FTC/TDF, coupled with a potential lower intrinsic risk of renal disease with EFV, result in a reduced frequency of clinically significant renal or bone adverse outcomes with EFV/FTC/TDF when compared with other TDF-containing regimens. Therefore, the purpose of this analysis was to compare the real-world incidence of renal and bone adverse outcomes among patients receiving different TDF-containing STRs.

## Methods

### Ethics statement

This study was conducted in accordance with International Society for Pharmacoepidemiology Guidelines for Good Pharmacoepidemiology Practices and applicable regulatory requirements.

### Study design

This was a retrospective analysis of two Truven Health MarketScan^®^ databases, the Commercial Claims and Encounters Database and Medicare Supplemental Database, using data extracted from the years 2008 to 2014. These databases include individuals and their dependents from all geographic regions in the US with commercial employer-sponsored insurance (Commercial Claims and Encounters Database; mainly individuals employed by large companies) and retirees with Medicare supplemental insurance paid by employers (Medicare Supplemental Database) [20]. In 2014, 62% of the US population was covered by either commercial employer-sponsored insurance or Medicare supplemental insurance [21]. The Truven Health MarketScan^®^ claims databases offer the largest convenience sample available in proprietary U.S. databases with more than 225 million unique patients since 1995 [20]. Thus, the data employed in this analysis can be considered to be representative of a commercially insured US population of patients with HIV infection.

STR exposure was defined as the first dispensing in the patient record of the index STR (EFV/FTC/TDF, RPV/FTC/TDF, or EVG/COBI/FTC/TDF), preceded by a 6-month baseline period with no dispensing of any antiretroviral drugs. Patients were followed-up until the first occurrence of one of the following events: 1) an outcome of interest; 2) end of exposure to the index STR, defined as the end of the days’ supply of the last prescription fill plus 30 days; 3) end of enrollment in the health insurance plan; and 4) end of the study period.

### Study population

Study inclusion criteria were as follows: 1) at least 1 medical record with a diagnosis of HIV-1 infection in adults aged ≥18 years during the identification period; 2) treatment with EFV/FTC/TDF, RPV/FTC/TDF, or EVG/COBI/FTC/TDF; and 3) ≥6 months continuous enrollment prior to commencing the index STR (baseline period). Study exclusion criteria were evidence of antiretroviral medication use in the baseline period and/or evidence of renal disease (for the renal analysis) or of bone disease (for the bone analysis) during the baseline period.

### Outcome variables

The primary outcomes of this study were the exposure-adjusted incidence rates (IRs) of renal and bone adverse outcomes by STR use.

Renal adverse outcomes were defined as at least 2 medical insurance claims that were associated with International Classification of Diseases, Ninth Revision, Clinical Modification (ICD-9-CM) diagnosis codes for renal disease according to an established algorithm [22], but with the exclusion of codes associated with calculus of the kidney and ureter. Bone adverse outcomes were defined based upon ICD-9-CM diagnosis codes for bone fracture according to an established algorithm designed to identify osteoporotic fractures [11]. For a detailed list of these codes, see S1 Table.

### Statistical analysis

For primary outcomes, the IR and 95% confidence interval (CI) assuming a Poisson distribution were reported by each STR. Comparison of the rates of adverse outcomes between EFV/FTC/TDF and RPV/FTC/TDF or EVG/COBI/FTC/TDF employed either IR ratios (IRRs), to estimate the relative risk for adverse outcomes between STRs, or IR differences (IRDs), to estimate the absolute excess or reduced rate of adverse outcomes attributable to use of EFV/FTC/TDF versus RPV/FTC/TDF or EVG/COBI/FTC/TDF at the population level. Multivariate Poisson regression analyses with adjustment for baseline measures were conducted, if the numbers of events of interest were of sufficient magnitude (otherwise, stratified analyses were performed), to evaluate the impact of treatment regimen on renal or bone adverse outcomes. To provide context (though not a specific study objective), IRs were also examined for all HIV patients in the database to determine if the rates observed among HIV patients receiving the STRs were markedly different from the overall HIV population.

## Results

### Renal and bone adverse outcomes for all HIV patients in the database

Among 126,168 HIV-positive patients within the database, renal adverse outcomes occurred in 5704 over 317,712 person-years of follow-up, representing an IR of 18.0 per 1000 person-years (95% CI: 17.5, 18.4).

Among 131,612 HIV-positive patients within the database, fracture occurred in 1710 over 393,857 person-years of follow-up, representing an IR of 4.4 per 1000-person years (95% CI: 4.2, 4.6).

### Renal adverse outcomes by STR

In the renal analysis, most demographic and baseline characteristics were balanced, with the following exceptions: a higher proportion of newer STRs were dispensed in later index years, and antibiotic and non-steroidal anti-inflammatory drug (NSAID) use were more frequent with RPV/FTC/TDF and EVG/COBI/FTC/TDF than with EFV/FTC/TDF (Table 1).

**Table 1:** Patient demographic and baseline characteristics in the renal analysis.

Characteristic	EFV/FTC/TDF (N = 8107)	RPV/FTC/TDF (N = 1017)	EVG/COBI/FTC/TDF (N = 752)
**Age, mean (SD)**	43.5 (10.5)	42.3 (10.9)	43.5 (10.8)
**Male, n (%)**	7026 (86.7)	854 (84.0)	669 (89.0)
**Calendar year, n (%)**			
2008–2009	2786 (34.4)	0	0
2010–2011	3473 (42.8)	248 (24.4)	0
2012–2013	1848 (22.8)	769 (75.6)	752 (100.0)
**Region, n (%)**			
North Central	1431 (17.7)	130 (12.8)	102 (13.6)
Northeast	1199 (14.8)	169 (16.6)	148 (19.7)
South	3879 (47.8)	460 (45.2)	333 (44.3)
West	1510 (18.6)	245 (24.1)	159 (21.1)
Unknown	88 (1.1)	13 (1.3)	10 (1.3)
**Hypertension, n (%)**	1772 (21.9)	241 (23.7)	192 (25.5)
**Cardiovascular disease, n (%)**	166 (2.0)	19 (1.9)	15 (2.0)
**Diabetes, n (%)**	679 (8.4)	90 (8.8)	81 (10.8)
**Hepatitis C virus infection, n (%)**	282 (3.5)	36 (3.5)	33 (4.4)
**History of substance abuse, n (%)**	120 (1.5)	28 (2.8)	20 (2.7)
**Antibiotic use, n (%)**	5602 (69.1)	833 (81.9)	619 (82.3)
**Prescription NSAID use, n (%)**	3628 (44.8)	607 (59.7)	463 (61.6)
**Hospitalization, n (%)**	1152 (14.2)	147 (14.5)	118 (15.7)

EFV/FTC/TDF: efavirenz/emtricitabine/tenofovir disoproxil fumarate; EVG/COBI/FTC/TDF: elvitegravir/cobicistat/emtricitabine/tenofovir disoproxil fumarate; NSAID: non-steroidal anti-inflammatory drug; RPV/FTC/TDF: rilpivirine/emtricitabine/tenofovir disoproxil fumarate; SD: standard deviation.

Within the EFV/FTC/TDF group, the number of patients and duration of follow-up were substantially greater than for the RPV/FTC/TDF and EVG/COBI/FTC/TDF groups (Table 2). Observed IRs for renal adverse outcomes with each STR were lower than the IR for all HIV patients in the database (Table 2).

**Table 2:** Incidence rates for renal adverse outcomes by STR.

STR	EFV/FTC/TDF (A)	RPV/FTC/TDF (B)	EVG/COBI/FTC/TDF (C)	All HIV patients
**N**	8107	1017	752	126,168
**Cases**	219	19	14	5704
**Person-years**	22,677	1812	1028	317,712
**Mean follow-up time, y**	2.8	1.8	1.4	2.5
Incidence rate (95% CI)*	9.7 (8.5, 11.0)	10.5 (6.7, 16.4)	13.6 (8.1, 23.0)	18.0 (17.5, 18.4)
**Crude IRR (95% CI)**				
A vs. B	0.92 (0.58, 1.47)	Reference		
A vs. C	0.71 (0.41, 1.22)		Reference	
**Adjusted IRR (95% CI)**†				
A vs. B	0.94 (0.59, 1.51)	Reference		
A vs. C	0.86 (0.50, 1.49)		Reference	
Crude IRD (95% CI)*				
A vs. B	−0.83 (−3.05, 1.15)	Reference		
A vs. C	−3.96 (−7.31, −1.06)		Reference	
Adjusted IRD (95% CI)*†				
A vs. B	−1.05 (−2.90, 0.53)	Reference		
A vs. C	−1.78 (−2.19, −1.50)		Reference	

*Per 1000 person-years.

CI: confidence interval; EFV/FTC/TDF: efavirenz/emtricitabine/tenofovir disoproxil fumarate; EVG/COBI/FTC/TDF: elvitegravir/cobicistat/emtricitabine/tenofovir disoproxil fumarate; IRD: incidence rate difference; IRR: incidence rate ratio; RPV/FTC/TDF: rilpivirine/emtricitabine/tenofovir disoproxil fumarate; STR: single-tablet regimen.

^†^Adjusted for age, sex, hypertension, cardiovascular disease, diabetes, hepatitis C virus infection, a history of substance abuse, antibiotic use, prescription NSAID use, and hospitalization (calendar year was not included as a covariate owing to strong collinearity with STR use).

**Fig 1:**
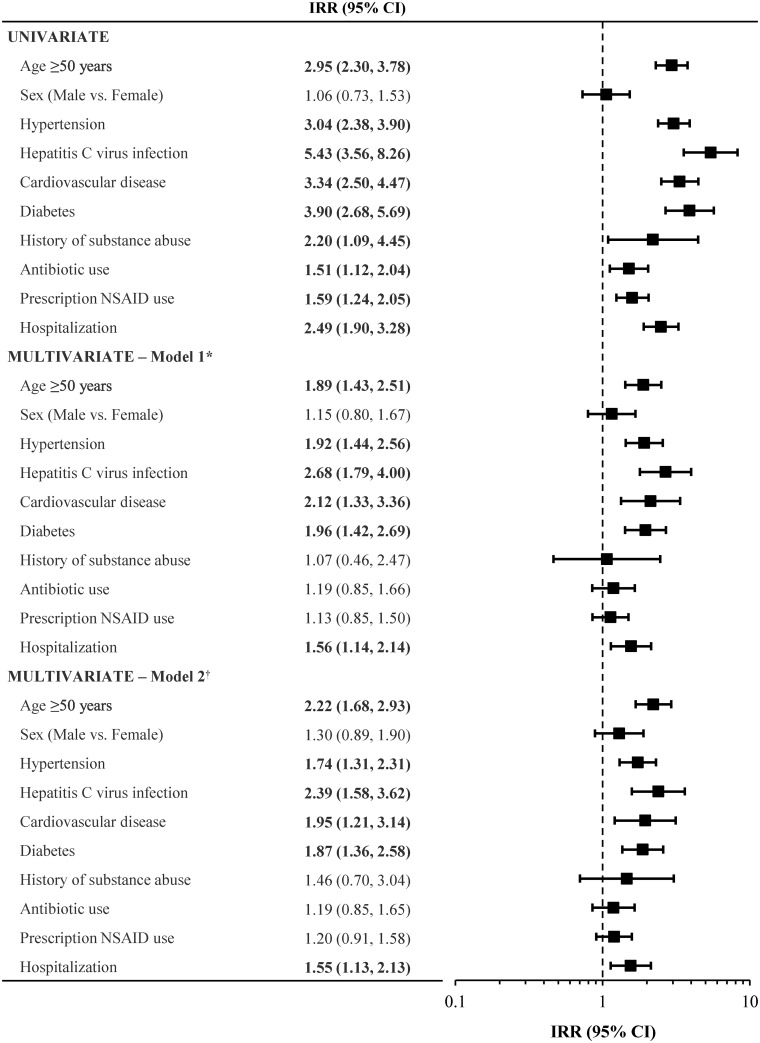
Univariate and multivariate adjusted IRRs (95% CI) for risk factors for renal adverse outcomes among patients prescribed STRs. *Including adjustment for EFV/FTC/TDF vs. RPV/FTC/TDF. †Including adjustment for EFV/FTC/TDF vs. EVG/COBI/FTC/TDF. Calendar year was not included as a covariate owing to strong collinearity with STR use. Bold typeface indicates that the CI does not cross unity. CI: confidence interval; EFV/FTC/TDF: efavirenz/emtricitabine/tenofovir disoproxil fumarate; EVG/COBI/FTC/TDF: elvitegravir/cobicistat/emtricitabine/tenofovir disoproxil fumarate; IRR: incidence rate ratio; RPV/FTC/TDF: rilpivirine/emtricitabine/tenofovir disoproxil fumarate; STR: single-tablet regimen.

Among patients prescribed STRs, significant risk factors for renal adverse events identified on univariate analysis were older age, hypertension, cardiovascular disease, diabetes, hepatitis C virus infection, a history of substance abuse, antibiotic use, prescription NSAID use, and hospitalization (Fig 1). On multivariate analysis, a history of substance abuse, antibiotic use, and NSAID use were no longer associated with adverse renal outcomes (Fig 1).

For comparisons between STRs, the IRD indicated a significantly reduced rate of renal adverse outcomes with EFV/FTC/TDF versus EVG/COBI/FTC/TDF (Table 2). The IRR results were directionally similar, but none were statistically significant.

### Fracture by STR

In the fracture analysis, most demographic and baseline characteristics were balanced, with the following exceptions: A higher proportion of newer STRs were dispensed in later index years, and chronic glucocorticoid use was more frequent with RPV/FTC/TDF and EVG/COBI/FTC/TDF than with EFV/FTC/TDF (Table 3).

**Table 3:** Patient demographic and baseline characteristics in the fracture analysis.

Characteristic	EFV/FTC/TDF (N = 7797)	RPV/FTC/TDF (N = 1262)	EVG/COBI/FTC/TDF (N = 1324)
**Age, mean (SD)**	43 (10.6)	42 (11.0)	43 (11.1)
**Male, n (%)**	6768 (86.8)	1061 (84.1)	1172 (88.5)
**Calendar year, n (%)**			
2008–2009	2746 (35.2)	0	0
2010–2011	3073 (39.4)	253 (20.0)	0
2012–2013	1976 (25.3)	1008 (79.9)	1324 (100.0)
**Region, n (%)**			
North Central	1363 (17.5)	161 (12.8)	157 (11.9)
Northeast	1158 (14.9)	214 (17.0)	240 (18.1)
South	3711 (47.6)	570 (45.2)	633 (47.8)
West	1405 (18.0)	291 (23.1)	260 (19.6)
Unknown	160 (2.1)	26 (2.1)	34 (2.6)
**Hypertension, n (%)**	1707 (21.9)	308 (24.4)	347 (26.2)
**Cardiovascular disease, n (%)**	246 (3.2)	42 (3.3)	45 (3.4)
**Diabetes, n (%)**	636 (8.2)	123 (9.7)	138 (10.4)
**Hepatitis C virus infection, n (%)**	270 (3.5)	49 (3.9)	45 (3.4)
**Chronic kidney disease, n (%)**	199 (2.6)	22 (1.7)	37 (2.8)
**History of substance abuse, n (%)**	114 (1.5)	34 (2.7)	43 (3.2)
**Smoking n (%)**	536 (6.9)	116 (9.2)	124 (9.4)
Chronic glucocorticoid use*, n (%)	909 (11.7)	209 (16.6)	264 (19.9)

*Defined as at least 60 days of cumulative exposure within an 18-month period.

EFV/FTC/TDF: efavirenz/emtricitabine/tenofovir disoproxil fumarate; EVG/COBI/FTC/TDF: elvitegravir/cobicistat/emtricitabine/tenofovir disoproxil fumarate; RPV/FTC/TDF: rilpivirine/emtricitabine/tenofovir disoproxil fumarate; SD: standard deviation.

Within the EFV/FTC/TDF group, the number of patients and duration of follow-up were substantially greater than for the RPV/FTC/TDF and EVG/COBI/FTC/TDF groups (Table 4). The IR for fracture with EFV/FTC/TDF was lower than the IR for all HIV patients in the database, the IR with RPV/FTC/TDF was comparable, and the IR with EVG/COBI/FTC/TDF was higher than all HIV patients in the database (Table 4).

**Table 4:** Incidence rates for fracture by STR.

STR	EFV/FTC/TDF (A)	RPV/FTC/TDF (B)	EVG/COBI/ FTC/TDF (C)	All HIV patients
**N**	7797	1262	1324	131,612
**Cases**	78	9	15	1710
**Person-years**	23,204	2495	2080	393,857
**Mean follow-up time, y**	3.0	2.0	1.6	3.0
Incidence rate (95% CI)*	3.4 (2.7, 4.2)	3.6 (1.9, 6.9)	7.2 (4.4, 12.0)	4.4 (4.2, 4.6)
**Crude IRR (95% CI)**				
A vs. B	0.93 (0.47, 1.86)	Reference		
A vs. C	0.47 (0.27, 0.81)		Reference	
Crude IRD (95% CI)*				
A vs. B	−0.25 (−1.02, 0.44)	Reference		
A vs. C	−3.85 (−5.02, −2.78)		Reference	

*Per 1000 person-years.

CI: confidence interval; EFV/FTC/TDF: efavirenz/emtricitabine/tenofovir disoproxil fumarate; EVG/COBI/FTC/TDF: elvitegravir/cobicistat/emtricitabine/tenofovir disoproxil fumarate; IRD: incidence rate difference; IRR: incidence rate ratio; RPV/FTC/TDF: rilpivirine/emtricitabine/tenofovir disoproxil fumarate; STR, single-tablet regimen.

**Fig 2:**
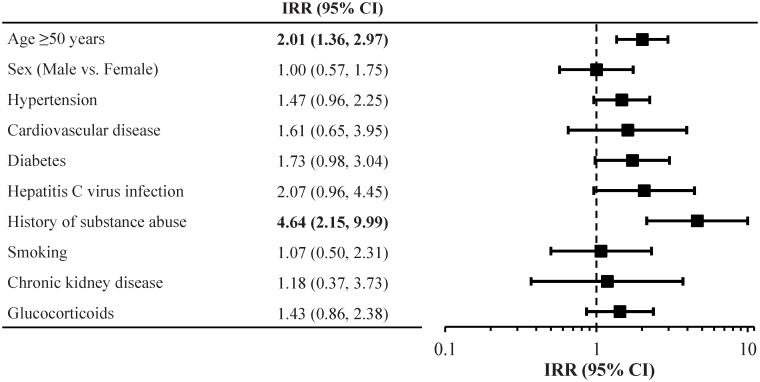
Univariate IRRs (95% CI) for risk factors for fracture among patients prescribed single-tablet regimens. Bold typeface indicates that the CI does not cross unity. CI: confidence interval; IRR: incidence rate ratio. *Defined as at least 60 days of cumulative exposure within an 18-month period.

Among patients prescribed STRs, significant risk factors for fracture identified on univariate analysis were older age and a history of substance abuse (Fig 2). Multivariate analysis could not be performed due to insufficient numbers of events. However, fracture IRRs for EFV/FTC/TDF versus RPV/FTC/TDF and EFV/FTC/TDF versus EVG/COBI/FTC/TDF stratified by risk factor were generally similar, indicating that risk factors for fracture did not vary by STR (S2 Table).

For comparisons between STRs, the IRR and IRD indicated a significantly reduced rate of fracture with EFV/FTC/TDF versus EVG/COBI/FTC/TDF (Table 4).

## Discussion

As hypothesized from the mode of administration and pharmacokinetic profile of EFV/FTC/TDF, IRs of renal adverse outcomes and fractures were lower with EFV/FTC/TDF than with EVG/COBI/FTC/TDF. Both cobicistat and rilpivirine increase serum creatinine (and hence decrease estimated glomerular filtration rate) via inhibition of renal tubular transporters [23], but this is not thought to affect actual glomerular filtration rate as measured by iohexol clearance [24]. Therefore, the renal and bone effects noted in the current analysis are more likely to be related to relative differences in tenofovir exposure across the three STR regimens evaluated. In this regard, the recommended administration of RPV/FTC/TDF and EVG/COBI/FTC/TDF with food increases tenofovir area under the concentration-time curve (AUC) by up to 38% [14] and 24% [19], respectively, relative to fasting administration. However, the additional effect of cobicistat on increasing tenofovir exposures (maximum concentration by 55%, AUC by 23% and trough concentration by 25% based on a TDF-cobicistat drug interaction study) [25], which is thought to be due to cobicistat inhibition of intestinal P-gp-mediated efflux of TDF [26], may have contributed to the higher IRs of renal adverse outcomes and fractures observed with EVG/COBI/FTC/TDF in this analysis.

The renal findings presented in the current study are consistent with those from comparative randomized clinical trials in treatment-naïve patients with HIV infection receiving RPV/FTC/TDF vs EFV/FTC/TDF or EVG/COBI/FTC/TDF vs EFV/FTC/TDF. In a study comparing RPV/FTC/TDF vs EFV/FTC/TDF, renal safety findings up to 96 weeks were similar across both regimens; 1 patient discontinued due to renal failure with RPV/FTC/TDF and 2 patients discontinued due to renal adverse events with EFV/FTC/TDF, (1 due to renal failure and 1 due to proteinuria and hypoproteinemia) [27]. In a study comparing EVG/COBI/FTC/TDF vs, EFV/FTC/TDF, renal safety findings up to 144 weeks appeared to favor EFV/FTC/TDF; 2.2% of patients discontinued due to renal adverse events with EVG/COBI/FTC/TDF (renal failure, Fanconi syndrome, increased serum creatinine, and abnormal glomerular filtration rate), whereas no patients discontinued due to renal adverse events with EFV/FTC/TDF [28]. Neither of the aforementioned clinical trials reported adverse bone outcomes.

In the current study, traditional risk factors such as older age, hypertension, cardiovascular disease, diabetes, and hepatitis C virus infection were significantly associated with renal adverse outcomes in this analysis. Having adjusted for the above factors on multivariate analysis, renal adverse outcomes were nevertheless significantly less frequent with EFV/FTC/TDF versus EVG/COBI/FTC/TDF (IRD: −1.78 per 1000 person-years; 95% CI: −2.19, −1.50).

Risk factors such as older age and substance abuse were significantly associated with fracture in this analysis, findings that are consistent with previous meta-analyses in patients with HIV infection or HIV/Hepatitis C viral coinfection [29,30]. Given the association between older age and substance abuse with reduced bone mineral density (BMD) and osteoporosis in the general population [31,32,33], the choice of antiretroviral regimen in patients with HIV infection with additional risk factors for osteoporosis needs to be carefully considered. Although the rarity of these events precluded multivariate analysis in the current report, on univariate analysis, fractures occurred significantly less frequently with EFV/FTC/TDF versus EVG/COBI/FTC/TDF (IRR 0.47; 95% CI: 0.27, 0.81 and IRD −3.85 per 1000 person-years; 95% CI: −5.02, −2.78).

Patient age, sex, and frequency of comorbidities at baseline were balanced across each STR group; therefore, differences in these characteristics were unlikely to have affected estimates of differences in adverse outcomes between STRs. However, chronic glucocorticoid use at baseline was more frequently observed with EVG/COBI/FTC/TDF than with EFV/FTC/TDF; the reason for this observation is unclear and is not possible to determine from this analysis. Although univariate or stratified analyses did not support an association with glucocorticoid use and fracture or an interaction between STR and glucocorticoid use for fracture, the inability to conduct a multivariate analysis for fracture precluded further investigation of a potential association and effect modification.

This analysis has limitations. First, the Truven Health MarketScan^®^ claims databases are based on a large non-randomized convenience sample of data mostly obtained from large employers; thus, the sample may contain biases and may not generalize to the wider population of patients with HIV infection (for example, patients with Medicaid cover or without insurance). Second, there is a possibility that adjustment for confounding factors, such as NSAID use, was insufficient in the renal multivariate analysis. Further, it was not possible to conduct a fracture multivariate analysis, so adjustment for confounding factors such as chronic glucocorticoid use could not be undertaken. Additionally, other factors that were unmeasured (e.g. baseline eGFR, CD4 count, viral load, other concomitant medication, co-morbidities) are potential confounders not accounted for by these analyses. Moreover, patients in earlier calendar years were likely to have been less healthy than those in later calendar years and likely to have initiated ART with lower CD4 count and higher viral loads. This could have led to an overestimation for the frequency of adverse outcomes with EFV/FTC/TDF, which had a higher proportion of patients starting treatment in earlier calendar years. Third, the low number of events (particularly for the fracture analysis) limited the precision of estimates; however, the estimated effects were consistent in terms of their direction and magnitude. As the observed fracture rate was expected to be low, to identify patients at higher risk of fracture but who had not yet experienced a fracture event, we conducted a separate bone adverse outcome analysis including patients with National Drug Codes for medications used for the treatment of osteoporosis. However, this mainly added a number of postmenopausal women to the database but did not increase the observed fracture rate to any significant degree. Therefore, it was not possible to determine whether the osteoporosis medications were being used as a result of treatment-associated decreases in BMD or increases in bone turnover, or for prophylaxis or treatment of female osteoporosis. Thus, in order to clearly evaluate potential associations between STRs and fracture without the potential confounding effect of osteoporosis medications, we present only the fracture analysis in this report. Fourth, although we employed an algorithm designed to favor capture of osteoporotic fractures, the methods employed in this analysis could not distinguish osteoporotic versus traumatic fractures with precision. Fifth, the mean duration of follow-up was relatively short, especially for the RPV/FTC/TDF and EVG/COBI/FTC/TDF groups. Sixth, owing to the increasing likelihood of initiating the newer STRs in later calendar years, the duration of follow-up with EFV/FTC/TDF was longer than with RPV/FTC/TDF or with EVG/COBI/FTC/TDF. This structural limitation could potentially lead to inaccuracies in the estimates of renal and bone adverse outcomes between the STRs included in this analysis. For example, the longer duration of follow-up with EFV/FTC/TDF is likely to be associated with an overestimation of the frequency of these adverse outcomes, assuming a certain lag time between the start of exposure to the antiretroviral drug and the onset of adverse outcomes. Instead, if the onset of adverse outcomes occurs early in the course of exposure to antiretroviral drugs and subsequently tails off (an unlikely scenario in this instance), this would be expected to lead to an overestimation for the frequency of adverse outcomes with RPV/FTC/TDF and with EVG/COBI/FTC/TDF. Ideally, prospectively designed studies employing delayed entry survival analysis could better adjust for the effect of changing patterns of antiretroviral use over time [34]. Finally, only renal and bone adverse outcomes were collected from the database and analyzed; thus, other side effects associated with the regimens included in the analysis, should be considered alongside renal and bone adverse outcomes when evaluating the overall risk:benefit profile of an antiretroviral treatment regimen.

A number of direct-acting antiviral drugs and drug combinations used to treat hepatitis C virus coinfection in patients with HIV, including simeprevir [35] and ledipasvir/sofosbuvir [36], can increase TDF exposure. However, comedications for hepatitis C virus infection were unlikely to have affected our results as the coinfection rate was balanced across the STR groups and was low (3.4–4.4%).

Tenofovir alafenamide (TAF), a novel pro-drug for tenofovir, is associated with a 91% lower plasma tenofovir concentration than that following TDF administration while maintaining high intracellular concentrations for HIV suppression [37]. Renal and bone biomarkers have shown more favorable changes with TAF-containing regimens than with TDF-containing regimens in a number of studies [38,39,40]. In the large GS-US-292-0109 study of patients switched from 1 of 4 TDF-containing regimens to the STR EVG/COBI/FTC/TAF, hip and spine BMD and glomerular filtration improved significantly in those switched to EVG/COBI/FTC/TAF compared with those remaining on a TDF-containing regimen [41]. However, serum creatinine increased the least in those remaining on EFV/FTC/TDF; and among the 4 TDF-containing regimens, reductions in BMD were numerically less in magnitude in those remaining on EFV/FTC/TDF than in those remaining on EVG/COBI/FTC/TDF (spine only) or atazanavir boosted with either COBI or ritonavir in combination with FTC/TDF (hip and spine) [41]. In recent prespecified subgroup analyses of the same study, patients switched to EVG/COBI/FTC/TAF versus those remaining on a TDF-containing regimen showed significantly greater differences in changes in proteinuria and tubular proteinuria favoring EVG/COBI/FTC/TAF [42,43,44]; however, these differences were numerically less in magnitude in patients switched from EFV/FTC/TDF to EVG/COBI/FTC/TAF [42] than in patients switched from EVG/COBI/FTC/TDF to EVG/COBI/FTC/TAF [43] or from boosted atazanavir plus FTC/TDF [44] to EVG/COBI/FTC/TAF. Whether the favorable biomarker changes with TAF-containing regimens will translate into corresponding reductions in adverse renal and bone clinical outcomes is currently unknown.

To conclude, in this large real-world database, observed IRs for renal adverse outcomes with TDF-containing STRs were lower or similar to those for all HIV patients. Among patients receiving STRs, the observed IR of renal adverse outcomes was lower with EFV/FTC/TDF than with EVG/COBI/FTC/TDF. Compared with all HIV patients, the observed IR of fracture was higher with EVG/COBI/FTC/TDF, was comparable with RPV/FTC/TDF, and was lower with EFV/FTC/TDF. Among patients receiving STRs, the IR of fracture was lower with EFV/FTC/TDF than with EVG/COBI/FTC/TDF. Despite containing TDF, EFV/FTC/TDF does not appear to increase renal adverse outcomes or fracture compared with a large population of HIV patients included in this analysis.

## Supporting Information

S1 Table ICD-9-CM codes. *Denotes all codes falling within the series.
(DOCX)

S2 Table Fracture IRRs for EFV/FTC/TDF vs. RPV/FTC/TDF and vs. EVG/COBI/FTC/TDF stratified by risk factor. CI: confidence interval; EFV/FTC/TDF: efavirenz/emtricitabine/tenofovir disoproxil fumarate; EVG/COBI/FTC/TDF: elvitegravir/cobicistat/emtricitabine/tenofovir disoproxil fumarate; IRR: incidence rate ratio; RPV/FTC/TDF: rilpivirine/emtricitabine/tenofovir disoproxil fumarate.
(DOCX)
