# Supplementary material for: Real-World Assessment of Renal and Bone Safety among Patients with HIV Infection Exposed to Tenofovir Disoproxil Fumarate-Containing Single-Tablet Regimens
Source: PLoS One. 2016 Dec 12;11(12):e0166982. doi: 10.1371/journal.pone.0166982 (PMC5152819; doi:10.1371/journal.pone.0166982)
Supplement: S1 Table — *Denotes all codes falling within the series. (DOCX) [file pone.0166982.s001.docx]

# S1 Table

**ICD-9-CM codes.**

| **Outcome measures** | **ICD-9-CM codes used** |
| --- | --- |
| **Renal** | 583*, nephritis and nephropathy (not specified as acute or chronic) |
|  | 584*, acute kidney failure |
|  | 585*, chronic kidney disease |
|  | 586, renal failure (unspecified) |
|  | 593.9, unspecified disorder of kidney and ureter |
| **Bone** | |
| **Vertebral fracture** | 805.2, closed fracture of dorsal (thoracic) vertebra without mention of spinal cord injury |
|  | 805.3, open fracture of dorsal (thoracic) vertebra without mention of spinal cord injury |
|  | 805.4, closed fracture of lumbar vertebra without mention of spinal cord injury |
|  | 805.5, open fracture of lumbar vertebra without mention of spinal cord injury |
|  | 805.6, closed fracture of sacrum and coccyx |
|  | 805.7, open fracture of sacrum and coccyx |
| **Hip fracture** | 820.0*, transcervical fracture, closed |
|  | 820.1*, transcervical fracture, open |
|  | 820.2*, pertrochanteric fracture of femur, closed |
|  | 820.3*, pertrochanteric fracture of femur, open |
|  | 820.8, closed fracture of unspecified part of neck of femur |
|  | 820.9, open fracture of unspecified part of neck of femur |
| **Wrist fracture** | 814.0*, closed fractures of carpal bones |
|  | 814.1*, open fractures of carpal bones |
|  | 813.4*, closed fracture of lower end of radius and ulna |
|  | 813.5*, open fracture of lower end of radius and ulna |
| *Denotes all codes falling within the series.  ICD-9-CM: International Classification of Diseases, Ninth Revision, Clinical Modification. | |
