# Supplementary material for: Real-World Assessment of Renal and Bone Safety among Patients with HIV Infection Exposed to Tenofovir Disoproxil Fumarate-Containing Single-Tablet Regimens
Source: PLoS One. 2016 Dec 12;11(12):e0166982. doi: 10.1371/journal.pone.0166982 (PMC5152819; doi:10.1371/journal.pone.0166982)
Supplement: S2 Table — CI: confidence interval; EFV/FTC/TDF: efavirenz/emtricitabine/tenofovir disoproxil fumarate; EVG/COBI/FTC/TDF: elvitegravir/cobicistat/emtricitabine/tenofovir disoproxil fumarate; IRR: incidence rate ratio; RPV/FTC/TDF: rilpivirine/emtricitabine/tenofovir disoproxil fumarate. (DOCX) [file pone.0166982.s002.docx]

# **S2 Table**

Fracture IRRs for EFV/FTC/TDF vs. RPV/FTC/TDF and vs. **EVG/COBI/FTC/TDF stratified by risk factor.**

|  | **IRR (95% CI)** | |
| --- | --- | --- |
| **Characteristic** | **EFV/FTC/TDF vs. RPV/FTC/TDF** | **EFV/FTC/TDF vs. EVG/COBI/FTC/TDF** |
| **Age ≥50 years** |  |  |
| Yes | 0.52 (0.22, 1.24) | 0.38 (0.17, 0.82) |
| No | 1.71 (0.57, 5.51) | 0.58 (0.26, 1.28) |
| **Sex** |  |  |
| Male | 0.77 (0.38, 1.55) | 0.47 (0.26, 0.85) |
| Female | Undefined | 0.45 (0.10, 2.00) |
| **Hypertension** |  |  |
| Yes | 1.34 (0.31, 5.68) | 0.40 (0.16, 0.99) |
| No | 0.83 (0.38, 1.82) | 0.52 (0.26, 1.06) |
| **Cardiovascular disease** |  |  |
| Yes | Undefined | 0.16 (0.03, 0.97) |
| No | 0.89 (0.45, 1.78) | 0.51 (0.29, 0.93) |
| **Hepatitis C virus infection** | | |
| Yes | 0.30 (0.06, 1.57) | Undefined |
| No | 1.11 (0.51, 2.42) | 0.44 (0.25, 0.76) |
| **Diabetes** |  |  |
| Yes | undefined | 0.43 (0.12, 1.53) |
| No | 0.79 (0.39, 1.58) | 0.49 (0.26, 0.90) |
| **Glucocorticoid use** |  |  |
| Yes | N/A | 0.73 (0.21, 2.54) |
| No | 0.71 (0.35, 1.43) | 0.43 (0.23, 0.80) |
| **History of substance abuse** | | |
| Yes | 0.90 (0.10, 8.02) | 0.43 (0.08, 2.35) |
| No | 0.98 (0.47, 2.03) | 0.50 (0.28, 0.90) |
| CI: confidence interval; EFV/FTC/TDF: efavirenz/emtricitabine/tenofovir disoproxil fumarate; EVG/COBI/FTC/TDF: elvitegravir/cobicistat/emtricitabine/tenofovir disoproxil fumarate; IRR: incidence rate ratio; RPV/FTC/TDF: rilpivirine/emtricitabine/tenofovir disoproxil fumarate. | | |
